# Asynchronous RTK Method for Detecting the Stability of the Reference Station in GNSS Deformation Monitoring

**DOI:** 10.3390/s20051320

**Published:** 2020-02-28

**Authors:** Yuan Du, Guanwen Huang, Qin Zhang, Yang Gao, Yuting Gao

**Affiliations:** 1 College of Geology Engineering and Geomatics, Chang’an University, 126 Yanta Road, Xi’an 710054, China; 2017026010@chd.edu.cn (Y.D.); zhangqinle@263.net.cn (Q.Z.); 2 Department of Geomatics Engineering, University of Calgary, Calgary, AB T2N 1N4, Canada; ygao@ucalgary.ca (Y.G.); yuting.gao1@ucalgary.ca (Y.G.)

**Keywords:** GNSS real-time kinematic positioning, reference station, stability, synchronous real-time kinematic positioning, asynchronous real-time kinematic positioning

## Abstract

The real-time kinematic (RTK) positioning technique of global navigation satellite systems (GNSS) has been widely used for deformation monitoring in the past several decades. The RTK technique can provide relative displacements in a local reference frame defined by a highly stable reference station. However, the traditional RTK solution does not account for reference stations that experience displacement. This presents a challenge for establishing a near real-time GNSS monitoring system, as since the displacement of a reference station can be easily misinterpreted as a sign of rapid movement at the monitoring station. In this study, based on the reference observations in different time domains, asynchronous and synchronous RTK are proposed and applied together to address this issue, providing more reliable displacement information. Using the asynchronously generated time difference of a reference frame, the proposed approach can detect whether a measured displacement has occurred in the reference or the monitoring station in the current epoch. This allows for the separation of reference station movements from monitoring station movements. The results based on both simulated and landslide monitoring data demonstrate that the proposed method can provide reliable displacement determinations, which are critical in deformation monitoring applications, such as the early warning of landslides.

## 1. Introduction

GNSS is one of several means of displacement monitoring and has been widely used for long-term stability analysis and the real-time dynamic monitoring of geo-hazards [1,2,3]. The most frequently used technique for deformation monitoring is RTK positioning based on double difference observations, where the relative position solutions are estimated with respect to a stable reference station [4]. The RTK technique can provide relative displacements with respect to a local reference frame defined by the reference station, which is crucial for the stability evaluations of landforms that become unstable as a result of geological hazards. Therefore, the stability of the deformation monitoring system reference station itself must be verified independently [5]. Otherwise, the displacement of the reference station could be easily misinterpreted as a sign of rapid sliding events occurring at the monitoring station.

To correctly interpret the deformation over time and space, it is necessary to establish and maintain a stable reference frame. This is accomplished with RTK positioning by a pair of valid observations from the same satellite, obtained simultaneously from both the reference station and the monitoring station, with the reference station being assumed to be a locally stable site. Because most spatiotemporal errors are reduced or eliminated by the differenced method, the RTK technique enables high-precision positioning in real-time applications. However, in many cases, the reference station can also be displaced by external influences, such as plate movements, fault motions, volcanoes, subsidence, hurricanes, and even human factors in urban environment [6,7]. Extensive research has been conducted to assess the stability of the reference frames [6,7,8,9]. Wang et al. [8] established a stable reference frame for landslide monitoring using five years of daily time series Global Positioning System (GPS) positions for the Puerto Rico and Virgin Islands region (using 24 h continuous GPS observations to calculate an average position). To develop post-mission single-site precise point positioning (PPP) deformation monitoring in the region, common points under the local and global frame were used to achieve a mutual conversion within the global reference frame IGS08 [8]. Bao et al. [6] not only realized a local reference frame, but also derived a regional seasonal model that can be applied to other deformation monitoring applications that require a stable reference frame. All of the previously developed reference frames have been based on the “absolute” positioning or PPP methods, which consider a global reference frame as being defined by the satellite orbits and clocks using a single GNSS receiver [10,11]. The disadvantage of PPP is that it requires a long convergence period of approximately 30 min to achieve centimeter-level accuracy (for high-frequency (1 Hz) GNSS RTK deformation monitoring applications, centimeter-level displacements may occur within 12 s) [12]. Despite the recent advances in accelerating the speed of PPP initialization with multi–GNSS), the process still requires at least 20 min [13,14,15]. Therefore, this method is often used for long-term, geological hazard monitoring based on low-rate, daily solutions [7,8]. Absolute gravimetry can also assess the vertical (but not horizontal) stability of any point on the surface of the Earth. However, it is considerably more expensive than GNSS [16,17]. Additionally, in applications of regional millimeter GNSS deformation monitoring, such as geological landslide monitoring, the general reference stations are distributed in the vicinity of the monitoring area (<5 km) in order to ensure real-time millimeter monitoring. Because most of the hidden landslide areas are located in mountainous areas, the height difference is large and the construction is difficult. So, the long-distance Continuously Operating Reference Station (CORS) network’s reference station cannot guarantee real-time millimeter monitoring accuracy. Considering the construction, cost, and stability of a landslide area, constructed regional reference stations are often unstable, so the stability of these stations must be monitored in real time. The framework network monitoring scheme for a large area and a long baseline is often used for post-monitoring, which is not suitable for stability detections of small-area reference stations in real time.

**Figure 1:**
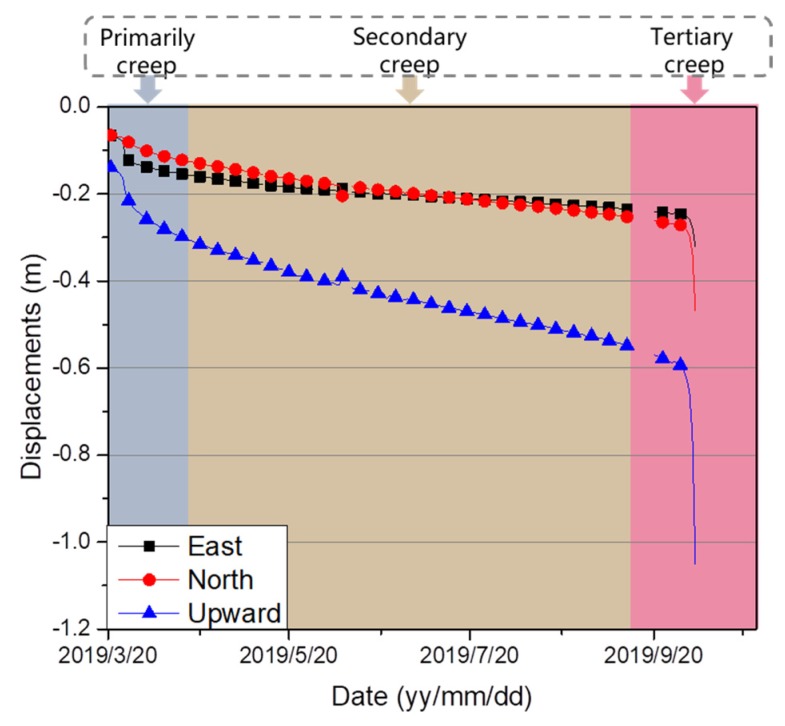
Conventional three-state interpretation of landslide creep behavior monitored at the Heifangtai landslide.

High-rate (e.g., a 1 Hz or higher frequency) GNSS positioning, however, is important for real-time displacement monitoring using the RTK method, such as with a tertiary (or accelerating) creep rate, increasing to that of a landslide, which can lead to a rupture [18]. Figure 1 shows the classic three-phase creep behavior of the Heifangtai landslide (located in Gansu Province, China), which indicates that, within a short time, a large displacement occurred in the vicinity of the final collapse, which occurred from 04:24 a.m. to 04:27 a.m. local time on 5 October 2019. Although reference stations are typically located in a stable area, this requirement can be difficult to achieve in practice. Reference stations may also be displaced during strong earthquakes [4,19]. Therefore, a stable reference frame must be maintained, even if for a short duration, for deformation monitoring and the rapid detection of when a reference frame shifts.

This study develops and adopts both asynchronous and synchronous RTK (ARTK and SRTK, respectively) methods to support real-time deformation monitoring at the centimeter scale, within a short period of time. Based on our previous study, the ARTK method can maintain centimeter-level positioning accuracy within 30 min [20]. In the presence of a shift in the reference frame for the current epoch, displacement information obtained via the SRTK method will become biased. In contrast, the displacement of the ARTK method will not be affected because of its reference frame being defined by the reference station of the epoch from a few minutes prior. In this manner, the proposed strategy of RTK positioning can overcome the influence of reference station displacement within a short time period. As a result, this strategy can detect and separate the displacement information of the monitoring station from the displacement information of the reference station, and finally provide more reliable displacement information. 

The remainder of this paper is organized as follows. First, the methodology of this new strategy using the SRTK and ARTK model is derived. Secondly, the experimental results using a simulated shift in the reference station are presented to validate the proposed method. The efficiency of this approach is also validated using 1 Hz GNSS data collected during the Heifangtai landslide creep monitoring, as shown in Figure 1. Finally, the conclusions drawn from this study are presented in the final section.

## 2. Methodology

The undifferenced observations for the code and carrier phase equations relating to satellite *i* and receiver *B* are as follows [21,22]:

(1) $$P_{B}^{i}\left(t_{0}\right)=\rho_{B}^{i}\left(t_{0}\right)+c\left[\delta t_{B}\left(t_{0}\right)-\delta t^{i}\left(T_{0}\right)\right]-I_{B}^{i}\left(t_{0}\right)+T_{B}^{i}\left(t_{0}\right)+E^{i}\left(t_{0}\right)+\varepsilon_{P,B}^{i},$$

(2) $$l_{B}^{i}\left(t_{0}\right)=\rho_{B}^{i}\left(t_{0}\right)+\lambda N_{B}^{i}\left(t_{0}\right)+c\left[\delta t_{B}\left(t_{0}\right)-\delta t^{i}\left(T_{0}\right)\right]-I_{B}^{i}\left(t_{0}\right)+T_{B}^{i}\left(t_{0}\right)+E^{i}\left(t_{0}\right)+\varepsilon_{l,B}^{i},$$

where P and l denote the code and carrier phase measurements, respectively; $t_{0}$ is the signal arrival time; $T_{0}$ is the corresponding signal emission time; ρ is the geometric distance from the satellite to the target; λ is the signal wavelength; N is the carrier phase ambiguity in cycles; c is the velocity of light; I is the ionospheric delay; T is the tropospheric delay; E is the ephemeris error; and ε denotes the measurement noise of the carrier phase and multipath.

The double difference observation removes biases, and a synchronous double difference observation model (which is applicable in the case of short baselines ≤10 km [23]) is used, as given by the following:

(3) $$P_{BA}^{ij}\left(t_{0}\right)=\rho_{BA}^{ij}\left(t_{0}\right)+\varepsilon_{P,BA}^{ij}\left(t_{0}\right)$$

(4) $$l_{BA}^{ij}\left(t_{0}\right)=\rho_{BA}^{ij}\left(t_{0}\right)+\lambda N_{BA}^{ij}\left(t_{0}\right)+\varepsilon_{l,BA}^{ij}\left(t_{0}\right)$$

Distinct from the synchronous model, some correlation errors cannot be eliminated in the following asynchronous double difference observation model [18,20]:

(5) $$\begin{array}{c} P_{BA}^{ij}\left(t_{0},t_{1}\right)=\rho_{BA}^{ij}\left(T_{0},T_{1}\right)+c\delta t^{ij}\left(T_{0},T_{1}\right)+E^{ij}\left(T_{0},T_{1}\right)+T_{BA}^{ij}\left(t_{0},t_{1}\right)\\ -I_{BA}^{ij}\left(t_{0},t_{1}\right)+\varepsilon_{P,BA}^{ij}\left(t_{0},t_{1}\right). \end{array}$$

(6) $$\begin{array}{c} l_{BA}^{ij}\left(t_{0},t_{1}\right)=\rho_{BA}^{ij}\left(T_{0},T_{1}\right)+\lambda N_{BA}^{ij}\left(t_{0},t_{1}\right)+c\delta t^{ij}\left(T_{0},T_{1}\right)+E^{ij}\left(T_{0},T_{1}\right)+T_{BA}^{ij}\left(t_{0},t_{1}\right)\\ -I_{BA}^{ij}\left(t_{0},t_{1}\right)+\varepsilon_{l,BA}^{ij}\left(t_{0},t_{1}\right). \end{array}$$

Because this method only considers centimeter-level displacement within a short period of time, the residual asynchronous errors can be ignored without further processing. Based on our previous study [20], the effects of other asynchronous errors (for example, the initial phase bias in the carrier phase measurement introduced by the satellite antenna) can be ignored, because these are relatively stable over a 15 min step size [24], and the errors introduced by the receiver antenna can be eliminated via the single difference method between satellites [20]; accordingly, they are not included in the equations. The tropospheric error is corrected using the Saastamoinen model and the asynchronous ionospheric delay is corrected using the dual-frequency carrier phase measurements from the current epoch of the monitoring station.

When the reference station is displaced, the difference between the coordinates of the monitoring station calculated separately by the SRTK and the ARTK methods reflects the displacement of the reference frame according to (where the term X represents the coordinate vectors of reference stations) the following:

(7) $$\Delta X\left(t\right)=X_{ARTK}\left(t\right)-X_{SRTK}\left(t\right)$$

When the reference station is stable, this difference is maintained at a consistent level. During this stable period, the proposed method determines the root mean square error (RMSE) of ΔX(t) . In this study, a value of ΔX(t) at more than three times the RMSE with a sustained growth trend over three consecutive epochs means that the fourth epoch is to be chosen as the start of the reference station drift. Correspondingly, the (t) in Equation (7) is considered to be the start time of the movement of the reference frame.

**Figure 2:**
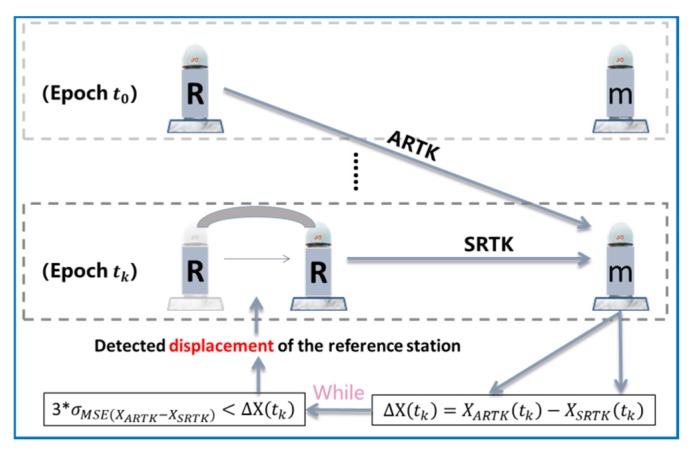
Synchronous real-time kinematic (SRTK) and asynchronous RTK (ARTK) models used to detect the displacement of the reference station (“R” represents the reference station and “m” represents the monitoring station).

The new strategy using the SRTK and ARTK models to detect the displacement of the reference station is illustrated in Figure 2.

Figure 2 shows that the two model solutions ($X_{ARTK}\left(t\right)\;\mathrm{and}\;X_{SRTK}\left(t\right)$) for the monitoring station can be obtained at every epoch. The reference frame of $X_{SRTK}\left(t\right)$ is based on the reference station of the current epoch, $t_{k\;},$ while the reference frame of $X_{ARTK}\left(t\right)$ is based on the reference station of the previous epoch, $t_{0}\;$. If the reference station shifts between epochs $t_{0}$ and $t_{k}$, this will cause a deviation in $X_{SRTK}\left(t\right)$. This is the displacement that must be detected. When using the ARTK algorithm to detect this deviation, there are two details that deserve further attention.

1. The accuracy of the ARTK model is significantly lower than that of the SRTK model [18]; hence, the difference between the two must be calculated by $\sigma_{MSE\left(X_{ARTK}-X_{SRTK}\right)}$ before setting the detection threshold. Thereafter, the ΔX(t) term obtained by each epoch will be compared with the previous statistical value of 3*$\sigma_{MSE\left(X_{ARTK}-X_{SRTK}\right)}$.
2. As the time between $t_{0}$ and $t_{k}$ increases, the positioning accuracy of the ARTK model is affected by the unresolved asynchronous error, producing a systematic deviation [20,22]. It is therefore important to note that this study only focuses on centimeter-level deformation shifts of the reference station over a short time. Therefore, the asynchronous step size set in this study does not exceed 1.5 min, and iteratively proceeds with a period of 1.5 min.

## 3. Data Analysis

To verify the proposed method, this study used the results obtained from simulated data and actual landslide monitoring data.

### 3.1. Simulated Displacement Monitoring

**Figure 3:**
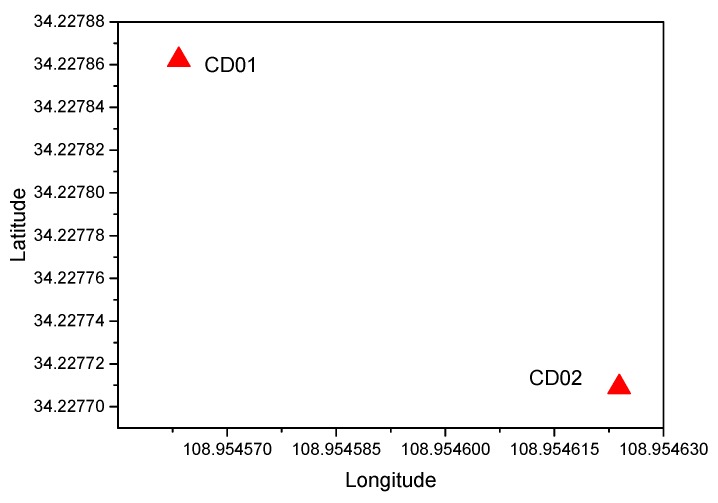
Distribution of stations CD01 and CD02.

**Figure 4:**
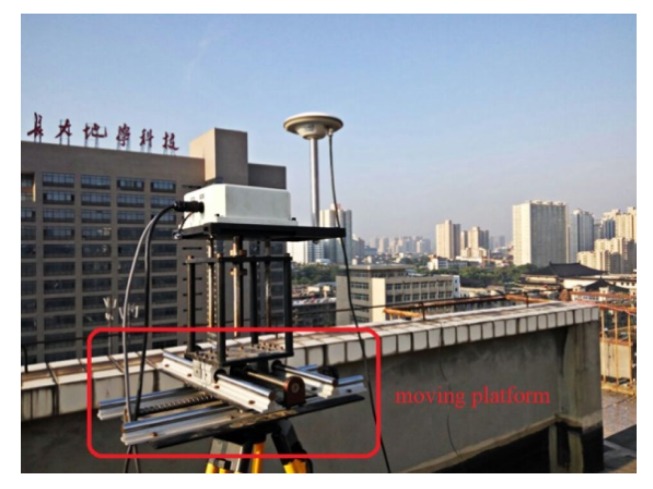
CD02 antenna installed on the moving platform.

**Figure 5:**
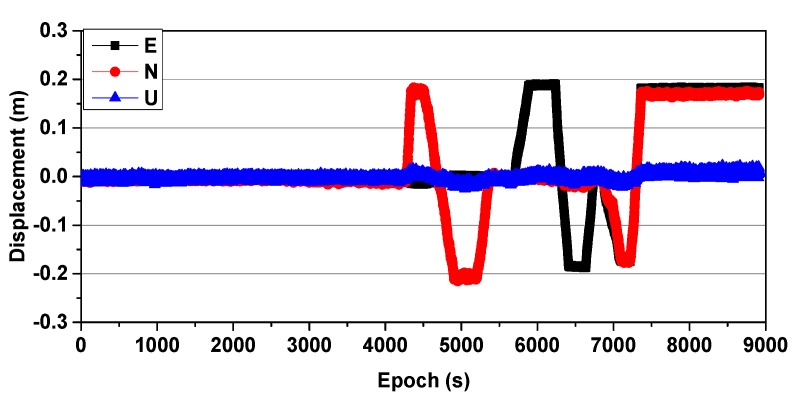
Displacement sequence obtained with CD01 as the reference station and CD02 as the monitoring station.

**Figure 6:**
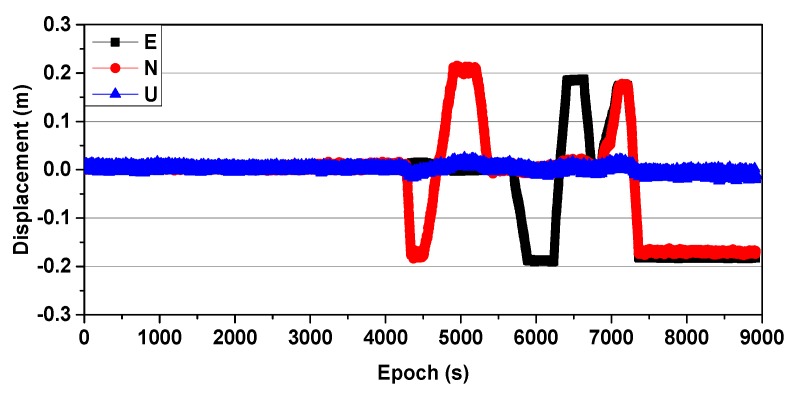
Displacement sequence obtained with CD02 as the reference station and CD01 as the monitoring station. E—east; N—north; U—upward.

Simulated GNSS (1 Hz) data were collected on 27 August 2016, and two receivers (Receiver UNICORECOMM-UR380 and Antenna HG-GOYH7151) were set up; these are referred to as CD01 and CD02, with the locations shown in Figure 3. Receiver CD01 was set up on a forced alignment pile and CD02 was set up on a moving platform to simulate slow displacement, as shown in Figure 4. First, CD01 was taken as the reference station and CD02 as the monitoring station, in order to obtain the displacement sequence diagrams shown in Figure 5 (where E, N, and U indicate displacement in the east, north, and upward directions, respectively), and the measured results were consistent with those applied in the simulation. Then, CD02 was taken as the reference station and CD01 as the monitoring station. Because the position of the reference station is usually assumed to be invariant in the calculation process, although CD01 was used as the monitoring station and did not have any displacement during the experiment, the displacement information of the reference station CD02 was expressed relative to CD01, and vice versa. The results showed that using CD01 as the monitoring station, even though it was fixed to an alignment pile without any displacement, and then moving CD02 as the reference station, resulted in an inverse but nearly identical measurement of movement compared to the results obtained when using CD01 as the reference station and CD02 as the monitoring station, as shown in Figure 6. Therefore, when the RTK technique was applied to displacement monitoring, it was particularly necessary to monitor the stability of the reference station.

**Figure 7:**
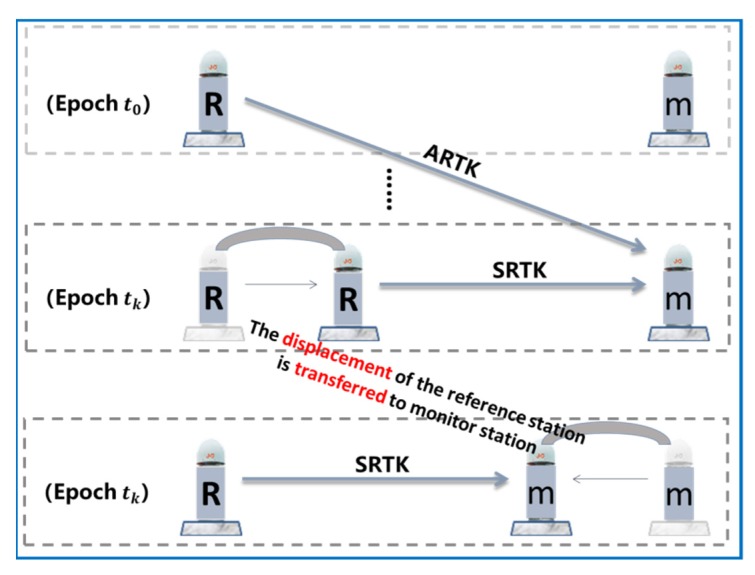
Simulated displacement of the reference station.

When the displacement of a reference station occurs, it can be easily misinterpreted as the movement of the monitoring station. As shown in Figure 7, the statuses of the reference station and the monitoring station in the topmost part (Epoch $t_{0}$) were known. The middle part corresponded to the epoch in which the reference station was shifted, and the traditional RTK technique was still used to obtain the position information of the monitoring station. Because the traditional RTK method defaulted to assuming the reference station was in a fixed location, the observed displacement of the reference station was transferred to the monitoring station, as shown in the bottom part of the figure.

**Figure 8:**
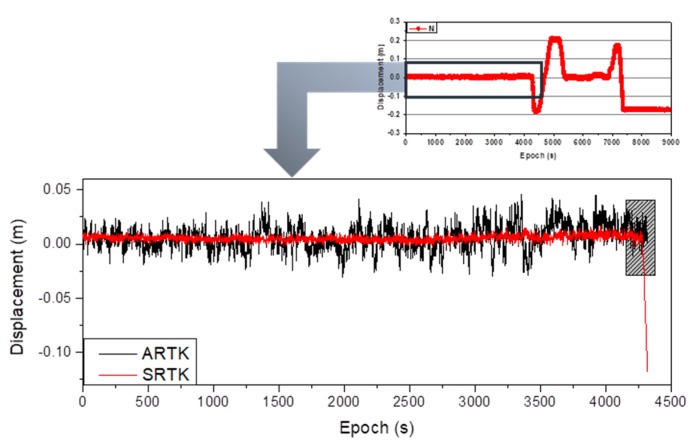
Epoch in which the displacement of the reference station is detected to exceed the threshold value.

**Figure 9:**
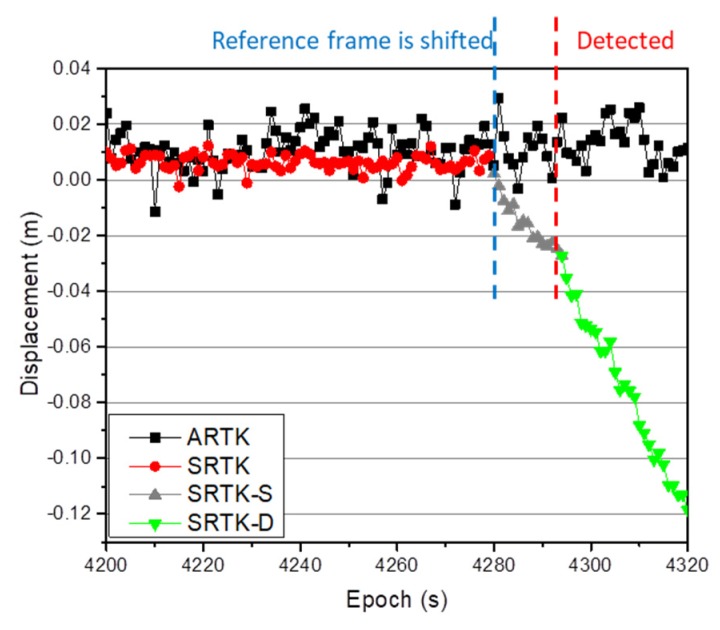
Enlargement of the epoch in which the displacement of the reference station is detected to exceed the threshold value.

To simulate the displacement of the reference station in the next experiment, CD02, which was installed on the moving platform, was selected as the reference station, and CD01 as the monitoring station. The positioning results in the N direction were then simultaneously calculated via the SRTK and ARTK methods, as shown in Figure 8. The figure shows that the positioning results obtained via the SRTK and ARTK methods remained in a stable range when reference station CD02 remained stationary. The $3\times \sigma_{MSE\left(X_{ARTK}-X_{SRTK}\right)}$ term was calculated according to the methodology in Section 2, and was determined to be equal to 0.035 m, to be used as the threshold for an acceptable difference between positioning results obtained via the STRK and ARTK methods. When the reference station was stationary, the difference between the results obtained for any three consecutive epochs via the two methods did not exceed this threshold. However, when reference station CD02 shifted in the N direction, the difference between the results obtained via the two methods increased significantly, as shown at the end of Figure 8. This part of the plot is enlarged and is shown as Figure 9.

Figure 9 indicates that the first epoch in which the reference station began to shift was epoch 4280. The grey phase in the figure is called the suspected (SRTK-S phase) reference station drift phase in this study, which was less than the defined threshold of $3\times \sigma_{MSE\left(X_{ARTK}-X_{SRTK}\right)}$. This phase lasted until epoch 4294, or 15 s. From epoch 4295 onwards, the difference between the results obtained via the STRK and ARTK methods was continually greater than the threshold value, and the reference station had shifted. The green phase in the figure is called the detected (SRTK-D phase) reference station drift phase in this study. Because the accuracy of the ARTK technique was lower than that of the SRTK technique, there was a stage during which the displacement of the reference station was less than the threshold prior to the final determination of the reference station status. The duration of this stage was not only related to the accuracy of the ARTK method, but also to the drift speed of the reference station. This duration was short for a higher accuracy ARTK technique and for a higher speed reference station shift. To improve the timeliness of the scheme in detecting the shift of the reference station, it was necessary to improve the positioning accuracy of the ARTK technique under the condition that the deformation state of the reference station remained constant. As mentioned in the literature [20], increasing the sampling rate of the clock difference effectively improved the positioning accuracy of the ARTK technique, so to accomplish this objective.

### 3.2. Landslide Monitoring

**Figure 10:**
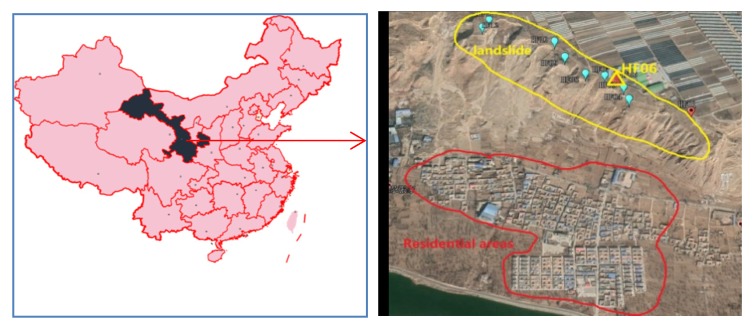
Location of the Heifangtai landslide.

To further demonstrate the validity of the proposed method, dual-frequency BeiDou data with a sampling interval of 1 s were collected from the monitoring of the Heifangtai landslide in Gansu Province, China. The average height difference between the top and bottom of the terrace was greater than 70 m, and the horizontal distance between the main body of the landslide and the main residential areas located beneath was approximately 400 m [25], as shown in Figure 10. Similar to traditional RTK monitoring, we placed a reference station in an area far away from the landslide (which was considered stable) in order to provide a relative reference for the monitoring point on the landslide [2].

**Figure 11:**
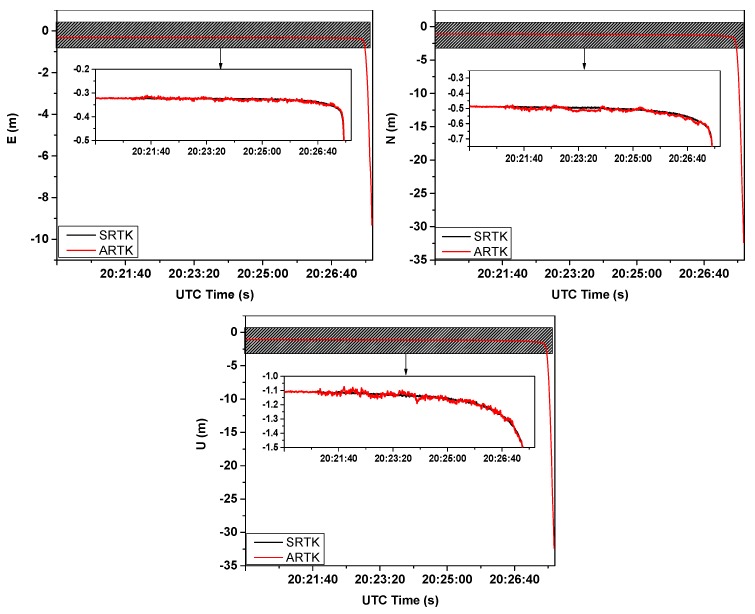
Consistency between the ARTK and SRTK methods in the E, N, and U directions during the final collapse of the landslide monitoring station.

**Figure 12:**
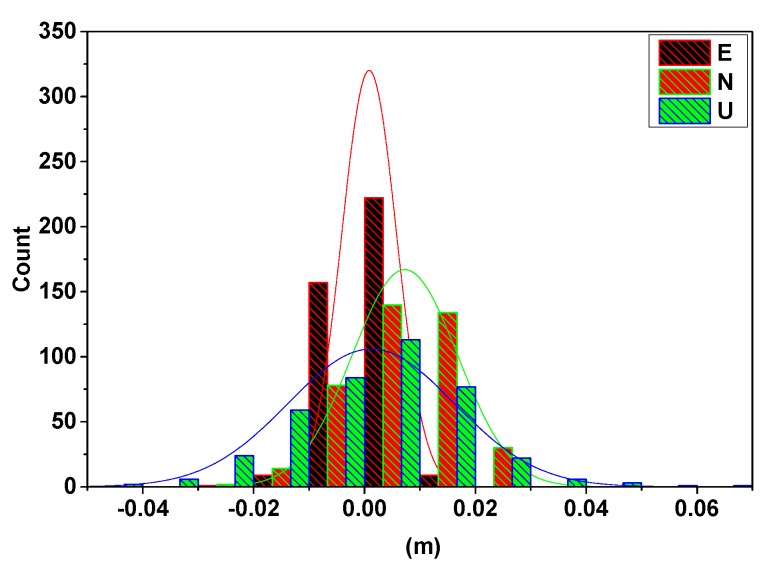
The counts of the difference between the ARTK and the SRTK techniques.

Section 3.1 describes the simulated experiment, which verified the effectiveness of the scheme when the reference station shifted. In this section, we will verify the consistency between the ARTK and SRTK techniques when the monitoring area is in a particular behavioral period, such as the tertiary creep of a landslide. The final collapse of the monitoring station (HF06) occurred from 20:24 p.m. to 20:27 p.m. (UTC) on 5 October 2019. Figure 11 shows the resulting displacements obtained in the E, N, and U directions via the SRTK and ARTK methods during that period. The slide was moved southwest at that time. The detailed numerical counts of the differences between the ARTK and the SRTK are shown in Figure 12.

To clearly compare the sequence of values obtained from the SRTK and ARTK methods, the shaded parts of the three plots in Figure 11 are shown as enlarged. The vertical direction demonstrates the first displacement trend, which is an observation that can aid in monitoring, predicting, and providing timely warnings regarding future landslide deformations. As the dilution of precision in this direction was substantial, the error was projected more in the height component than that in the E and N directions. However, it was still within a stable range. The most accurate results were obtained in the E direction, which benefitted from the geometry of the five geosynchronous Earth orbit satellites of the BeiDou system, stationed over the Asia–Pacific region from the west to the east [22]. Figure 11 shows that when the SRTK and ARTK methods were applied to the imminent sliding stage of the landslide, the displacement sequences of the two methods remained consistent and within the threshold. The difference between the ARTK and the SRTK was concentrated around 0 m, as shown in Figure 12. This consistency proved that the displacement during this period was only the result of the monitoring station movement and that the reference station did not shift. Therefore, even in the special case of the final collapse of a landslide, the ARTK and SRTK techniques can be simultaneously used to monitor the landslide and to determine whether the displacement comes from the reference or monitoring station. In this manner, the proposed method can provide reliable high-precision deformation information, which is critical in geo-hazard mitigation applications, such as providing early warnings of impending landslides.

## 4. Conclusions

In summary, a new strategy was proposed and evaluated in this study. The historical data from a reference station without relative displacement was used to construct the ARTK observation model. The asynchronously generated time difference was used to obtain the positioning results from the SRTK and ARTK techniques, based on the location of the reference point in different time domains; this can aid in detecting whether the reference station has been displaced in the current epoch. The experiments conducted using a simulated reference station shift showed that when the cumulative displacement of the reference station exceeded the selected threshold, it was detected in time, without misjudging the movement within the monitoring area. The results obtained using real-time monitoring data from the Heifangtai landslide in Gansu, China, showed that the proposed algorithm can provide reliable deformation information so that informed decisions can be made regarding safety, even if a landslide is undergoing an imminent final collapse. Thus, the proposed approach effectively overcomes the limitations of the existing techniques with regard to determining the stabilities of the reference stations within a short period of time, and provides effective and reliable displacement data to enable accurate predictions and early warnings regarding the deformation area. In conclusion, the proposed technique has various merits with regard to the application and promotion of the RTK method in the field of geological disaster monitoring.
